# Best Method of Anesthetizing Children

**Published:** 1907-07

**Authors:** 


### SELECTIONS AND ABSTRACTS.

### BEST METHOD OF ANESTHETIZING CHILDREN.

There seems to be no consensus of opinion on the subject. Dr. S. J. Kopetsky, anesthetist to the Harlem Hospital, New York, combats the idea that chloroform is the best anesthetic for children. He says that cardiac failure may set in at the very beginning of the anesthesia, when the children begin to struggle. Death may occur suddenly, without warning. Ether is less dangerous, but the best method of anesthetizing children is by the aid of nitrous oxide and ether. By preceding ether with nitrous oxide a less amount of ether is required, the after-effects thus being diminished.—**Medical Times.**

